# Recherches de Pathologie Comparée

**Published:** 1846-04

**Authors:** 


I. Recherches de Pathologie Comparée. Par *Ch. F. Heusinger*. 4to. Pp. 700. Cassel, 1844.

II. The Veterinarian, from January to December, 1845.

The work of M. Heusinger is one of extraordinary merit, from the vast amount of curious literary and scientific information which it contains. The subject, or rather subjects, of which it treats, although not immediately appertaining to human medicine, ought assuredly not to be entirely overlooked by the professional reader. Many of our ablest medical writers have not deemed the study of Zootic diseases unworthy of their notice. As far back as the 16th century, we find that a work was published by Ingrassias at Venice, entitled *Veterinaria medicina formaliter una eademque cum nobiliori hominis medicina, materiæ duntaxat nobilitate differens*. Towards the close of last century, Franck published an excellent essay *de morbis pecudum a medentibus nequaquam prætervidendis;* and, during the first ten years of the present one, several works were brought out in France upon this very interesting branch of pathological research. More recently, we have the distinguished names of Rayer and Andral to point to as authorities for its importance. The former, amid the multiplicity of his engagements, has found time to start a journal upon the very subject of comparative medicine; and the latter has been of late extending, we are glad to find, his valuable researches, on the morbid conditions of the blood in different diseases, from man to the lower animals. It must be quite unnecessary to say more in the way of apology or recommendation; if we required any argument, we have only to utter the simple name of Vaccination: *that* will carry more weight and conviction than the most elaborate reasoning, or a host of the most distinguished authorities.

"The physical life of man," M. Heusinger remarks, "having the same conditions, and exhibiting the same general phenomena as that of animals, must also present the same resemblance in the state of disease. We may, therefore, very properly ask, what influence has the medicine of animals upon that of man? and what influence ought it to have?" It may not be possible to determine the exact amount of such influence; but this at least may be very reasonably asserted, that the study of the diseases of animals—more especially those of a contagious, and of an endemic and epidemic, perhaps we should rather say enzootic and epizootic nature—promises to reflect considerable light upon some important points not only in human pathology, but also in general hygiene.

Our author's work contains a variety of elaborate essays, or treatises, upon different subjects connected with Comparative Pathology. The first of these is a history of Veterinary medicine from the earliest times down to the present day. The fine scholarship, as well as the unwearied research, displayed in this paper, cannot but interest the reader.—The second is entitled a "Comparison of the diseases described by the ancient writers on veterinary medicine, and by those of the middle ages." The medical antiquary, more especially, will find much to engage his attention here.—The third, is an "Historical view of the doctrine of the diseases of Birds."—The fourth, is called a "Sketch of a comparative Nosography of man and of domestic animals." The object of this most valuable and erudite treatise is to prove that very nearly the same diseases occur in veterinary as in human medicine; and, therefore, that the study of the one is calculated to throw much light upon that of the other. The amount of professional information exhibited upon this interesting subject is immense.—The fifth, and last, is entitled "Diplomatic Chronology of Epizootic Diseases." This chronological history commences with Homer's celebrated description of the plague among both man and beast, which Apollo sent into the Grecian camp at the siege of Troy, and is carried down, step by step, through classic times to the Christian Æra, and thence through succeeding centuries to the wide-spread and destructive apthongular Epizootic which prevailed throughout Europe from the year 1838 to 1842. It is called "diplomatic," from the circumstance that it describes, *pari passu*, the epidemics that existed cotemporaneously with the Epizootics whose histories are related. It is altogether a wonderful performance. We had hoped to have been able to give a brief review of it; but we are unwilling to occupy too much space with matters which may be considered as merely accessary, and of only secondary professional importance. We shall, therefore, in the present number, confine our notice entirely to an analysis, imperfect indeed, of the fourth paper—the Comparative Nosography of Man and Animals.

The subject is more immediately interesting, and is calculated to excite the attention of medical men generally, and especially of those who reside in the country, or, from their connection with our army, may be called upon to visit foreign lands.

We begin our notice with the

DISEASES OF THE DIGESTIVE APPARATUS.

*Stomatitis Aphthosa**—characterised by the eruption of aphthæ, degenerating into ulcers, in the cavity of the mouth, and accompanied with fever—is of common occurrence in all our domestic animals, more especially among the ruminants. It appears to be contagious in certain Epizootics. Sometimes it attacks only one tribe of animals; at other times, scarcely any is exempt. Not unfrequently the human species, in infancy and childhood more especially, is affected at the same time. Occasionally it has been observed among poultry.

A gangrenous form of Stomatitis, affecting more particularly the tongue, sometimes prevails among domesticated animals, especially pigs. It has been described under the names of "cancro-volante," "glosso-anthrax," "la soie" (in the case of pigs), &c. The disease in birds, which has been called Anthrax of the throat, appears to be similar to gangrenous or putrid Angina in man; and some of the Epizootics of *chancre volant* in cattle seem to have been of the same nature.

There is a disease, which consists in a prolongation of the mucous follicles of the cheeks and tongue, and described by the Italian and French writers under the names "barbole" and "barbillous," that is met with in horses and oxen. We are not acquainted with anything exactly similar to it having been observed in man; although the tongue itself, or its papillæ, are sometimes very considerably hypertrophied. The papillary hypertrophe seems to be that disease which the German people call "Zungenwurmer." All the forms of *Angina* have been observed in the lower animals; but we want exact information as to the comparative liability of different tribes of animals to the disease.

*Warts on the tongue* are very rarely met with in the human subject; but they are common in the horse, and still more so in the dog, in consequence of the greater development of the epithelium.

The first and second *Dentitions* are dangerous periods for animals as well as for mankind. If we are to believe Shaw, the traveller in Barbary, not a few young lions die from this cause. Certain it is that the greater

---

* The genuine *stomatitis pultacea* vel *pseudo-membranacea*—the *muguet* of French writers—characterised by the formation of membranous exsudations on the surface of the cavity of the mouth, has not been observed in any of the lower animals.

number of orang-outangs die off in captivity, during the period of dentition. Many of our domestic animals, horses and dogs for example, suffer a great deal at this period.

*Parotitis*, or inflammation of the parotid glands, is not unfrequently an epizootic, as well as an epidemic, disease.

*Salivary lithiasis*, or the formation of calculi in the salivary ducts, is more common in some of the lower animals than in man.

*Vomiting* may be regarded as a natural or physiological act in the dog and sow. When inconvenienced by any food that has been swallowed, these animals are in the habit of rejecting, and again swallowing, it. In ruminants, on the other hand, the occurrence of vomiting always indicates some grave disease of the stomach; in the horse it is very generally a mortal symptom.

M. Heusinger remarks, in reference to *Dyspepsia* in animals, that "inaction of the first stomach is often observed in ruminants, and that the benefit derived from the internal use of muriatic acid is very striking."

*Pneumatosis* or excessive *Tympanitis* of the paunch, from the immoderate use of young succulent herbs, is a common, and often very dangerous, disease in cows and sheep. Besides carbonic acid, hydrogen and carburetted hydrogen are sometimes formed in immense quantity within the cavity of the stomach.

The *rosio vaccarum*, or the tendency to lick everything—especially saline, calcareous, or earthy substances,—is always the sign of a cachexia, and may be compared to the *pica* in chlorotic girls.

*Gastritis* occurs frequently in all our domestic animals; but its various forms have not hitherto been well distinguished by veterinary writers. Mucous gastritis is common and very dangerous among dogs. A dangerous form of mucous gastro-enteritis is often met with in horses, and especially in ruminants, after they have been feeding in forests. Inflammation of the serous coat of the stomach is apt to occur in these animals, after chills.

*Perforations of the stomach* by ulcers and mortification are not unfrequently found in dogs and horses; but we are not aware that any appearance exactly similar to the *gastro-malacia* or gelatiniform softening, that is apt to occur in young children, has been ever observed; although solution of the stomach after death is frequently noticed in rabbits and other animals.

*Lithiasis gastrica.*—In the human subject, biliary concretions have sometimes, though very rarely, been discovered in the stomach. As to gastric calculi of any other sort, we have no satisfactory evidence that such have ever been found. In the stomach of the dog, however, and still more frequently in that of the horse, large calculi have been often met with. Their mode of formation is not quite understood. They usually contain a little silica. In the case of ruminants, they seem to be formed by a mass of hairs, agglutinated together.

*Entozoary worms* have often been observed in the stomach of domestic animals. The Spiroptera sanguinolenta and the Strongylus trigonocephalus have been found in the dog; the Spiroptera megastoma in the horse; the Strongylus contortus in the sheep: the Spiroptera strongylina in the sow; and the Amphistoma conicum in cows and sheep. Helminthiasis is more frequent in all domestic animals than in the human species; and the number of worms present is sometimes enormous. Muyschel has recorded a case of complete and fatal obstruction of the intestinal canal in the horse by a mass of lumbrici. Different species of the Ascaris, Tænia, Strongylus, Echinorhyncus, Distoma, Botriocephalus, Oxyuris and Trichocephalus, are met with in the horse, cow, sheep, sow, dog and cat. Some of these worms in the lower animals are more dangerous to life than those found in man.

*Enteritis* is a common disease among animals. In them, it is more frequently combined with genuine gastritis than in man. Veterinary writers have not discriminated with sufficient skill the mucous from the serous form of the disease. Much depends, unquestionably, upon the condition of the atmosphere, the action of the climate, and soforth. In low wet countries, there is an epizootic, as well as an epidemic, tendency to morbid affections of the mucous surface of the stomach and bowels. MM. Delafond and Bouley have recently published, in the Recueil de Med. Vet. t. xix, some interesting observations upon a form of enteritis, which they call "e. couenneuse."

*Lithiasis intestinalis* is of much more frequent occurrence in animals, more particularly the herbivorous, than in man. We distinguish the so-called "ægagropiles," formed by hairs agglomerated together, and incrusted or not, from the proper phosphatic calculi, the nucleus of which is usually formed by vegetable fibre, bran, or some other foreign substance. They sometimes attain so large a size as completely to obstruct the bowels and occasion death.*

---

* The following description of Intestinal Concretions in Animals is taken from Simon's Chemistry.

"They are by no means rare either in the carnivora or herbivora; they seem to be especially common in horses. They consist principally of the most insoluble salts that occur in the food, which, instead of being uniformly distributed throughout the whole of the chyle, are collected at particular points, or else, after having been dissolved in the stomach, are precipitated in the small intestines by the free alkali of the bile, and settle around any nucleus they may meet with. The principal constituents of intestinal concretions are phosphate of lime, phosphate of magnesia, ammoniaco-magnesian phosphate, and occasionally the carbonates of lime and magnesia. Gurlt remarks that the reddish gray concretions found in the stomachs of horses sometimes attain a very considerable size: (he mentions a case in which a concretion weighed 14 pounds); they consist of concentric laminæ, and are very hard. In horses they have occasionally a bluish-gray tint."

*Diarrhœa* is quite as common in young animals while teething, as in children during the same period.

*Dysentery* sometimes prevails, more especially in warm climates, as an Epizootic and Enzootic, as well as an Epidemic and Endemic, malady; it is often very destructive to the brute creation.

*Torsion*, or twisting *of the gut* is much more frequent in the horse than in the human being. In the latter, it almost always takes place in the flexure of the colon; in the former, it is usually in the free part of the colon or in the cæcum itself. The attack is generally fatal. It has been proposed to cut into the cavity of the abdomen, and unravel the twisted fold of intestine; but we are not aware whether the operation has been ever performed.

*Rupture* of the intestine is a not very unfrequent accident in cows and horses, being generally the result of obstinate constipation, the presence of calculi, or of a twisting of the tube. The lodgment of the fæces is, on the whole, more frequent, and is also apt to become a more serious affection, in some of our domestic animals than in the case of man.

Most of our domestic animals are liable to *prolapsus ani;* and even *fistula* in this part is not very rare among dogs and horses. Worms are much more frequently present in the large intestines in animals than in the human being. In the cavity of the abdomen too, hydatidic formations —especially Cysticerci and Echinococci—are more common.

Various species of *Hernia* are met with in the lower animals. Congenital umbilical hernia is almost as common among them as in man: it is most frequent in the pig. Although the inguinal canal remains permanently open in animals, hernia at this point is of exceedingly rare occurrence. The ventral species is much more frequent. Diaphragmatic hernia is common, especially in the horse: in many instances, probably, it is of congenital occurrence.

Different morbid affections of the Spleen appear to be far from uncommon among animals; more especially in hot and marshy climates. Indeed, wherever they are endemic, they are apt to be enzootic. This viscus is often found to be enormously distended with blood; at other times it is decidedly inflamed, or hypertrophied, or softened and reduced to a pulpy consistence, or occupied with tuberculous deposits or hydatidic formations. Occasionally it becomes ruptured. What we have said respecting the Spleen, is equally applicable to the Liver. There is a peculiar form of Acute Hepatitis, inducing excessive softening of the hepatic parenchyma, which often proves very fatal to sheep (hepatite typheuse, putride, lebertyphus). Hydatids and other entozoa are, it is well known, exceedingly common in these animals. The presence of "flukes" in the biliferous tubes very often gives rise to inflammation; and the result of the inflammatory action, especially in the goat, is often a remarkable sort of ossification. *Icterus* has been frequently noticed in the dog, swine, and sheep: it is rare in the horse.

*Cholo-lithiasis*, or the presence of Biliary Calculi, is much less common,

(although far from being rare) in the lower animals than in man. Their nature and composition too seem to be of a different character.* Those, that are found in the ox, have often a metallic lustre on their surface. The famous "pietra di porco" of India is a biliary calculus of the Erinaceus malacensis; and probably the bezoars of the East are often nothing but the gall-stones of some herbivorous animal.

### Diseases of the Respiratory Organs.

*Catarrhal Affections.*—There is often a striking resemblance between these in animals and in human beings. What is more particularly worthy of notice is, that in each species of animal, the malady exhibits some peculiar or specific character. Thus, in the catarrh of the *horse*, the lymphatic glands usually swell very quickly, and a scrofulous condition (etat gourmeux) is developed, which is apt to pass into a glanderous cachexy. In young persons, in whom there is a decided scrofulous or phthisical constitution, we have often occasion to observe that catarrh is apt to occasion swellings of the lymphatic glands, not unlike the "gourme" of the horse.

The catarrh of the *cow* passes quickly into an ulcerated and gangrenous state, which is usually highly dangerous.

In the *sheep*, the disease is apt to put on a cachectic, or even a putrid, character, constituting what has been called the "rot" in these animals. In the *dog*, it often assumes a nervous and ataxic character: this state is called by the Germans "hundestaupe."

All catarrhal diseases exhibit a marked tendency to become contagious, and to pass from one species of animal to another. They often prevail epizootically, especially among horses, when there is an epidemic of influenza among human beings. The verminous and pituitous condition of the lungs† in lambs, calves, and pigs often exhibits a striking analogy with the verminous and pituitous state of the bowels in children. These diseases attack the same age, are endemic and enzootic in the same countries (Holland, in the neighbourhood of Goettingen, &c.), and prevail epidemically and epizootically in the same seasons.

*Enlargement of the Thyroid Gland*, though not so common as in man, is far from being unfrequent in some animals—in the dog, for example. It is enzootic in those countries where it is endemic among the inhabitants.

### Cutaneous Diseases.

M. Heusinger remarks that the great differences between the human skin and that of animals must cause very considerable differences in their

* The biliary concretions of cattle differ considerably from those of man; they consist, for the most part, of biliary pigment and resin, with a little cholesterin.—*Simon's Animal Chemistry.*

† *Helminthiasis* of the air-passages has never been met with in the human subject, although it is of common occurrence in brutes: it is, as might be imagined, a very dangerous affection. Young animals appear to be most liable to it.

morbid affections. The freedom and mobility of the skin in animals, the development of the cutaneous muscle, the abundance of the cellular tissue, and the amplitude of the lymphatic system under the skin, will account for some of these differences. "It may be necessary indeed, considering these points of diversity, to designate certain cutaneous diseases in animals by peculiar names, differing from those used in human dermatology; but we trust that, whenever it is possible, the same terms will be used for analogous and corresponding morbid phenomena. He, who undertakes to describe the cutaneous diseases of animals, should well understand those of the human subject—which has not hitherto been the case with veterinary writers,—and he, who shall faithfully pourtray the development and metamorphoses of any one disease, will deserve better of science, than he who devises a dozen of new appellations."

*Erysipelas* is a not unfrequent epizootic and contagious disease among all our domestic animals. Its various species—*læve, vesiculosum, bullosum, pustulosum,* and *gangrenosum*—occur in them as in man. It is much more common in sheep and pigs, than in horses and dogs.

*Morbilli* occurs as a contagious, and often very dangerous, disease in sheep, and also in pigs; and cases have been recorded where the disease seemed to be communicated from the human subject to the lower animals.—*Scarlatina* also is recognised as a disease that has been observed in dogs, horses, and swine.—*Urticaria* is of frequent occurrence in almost all our domesticated animals.

Of papular eruptions, Prurigo is well known to be a very common Zootic disease; but its general and local forms have not yet been clearly distinguished.

*Scabies* occurs in all our domestic animals; and, in all, the scabious insect has been discovered to exist. Hertwig has proved by numerous observations the transmission of the disease, not only from one animal to another, but also from an animal to the human being. The facility of transmission, however, appears to be the greatest between animals of the same species.

Various forms of Ecthymatous, Impetiginous, and Porriginous eruptions are observed in most animals. Hitherto their true nature has not been ascertained with any degree of precision. Indeed, the whole subject of Cutaneous pathology, human as well as comparative, requires revision; there is so much confusion occasioned by applying the same terms to very different diseases. Greve has described, under the appellation of *porrigo decalvans,* a disease in horses similar to that observed in man; Haubener has very improperly designated it *herpes decalvans.*

*Variola.*—Our author regards the various forms of this Exanthem, from the simplest vesicular Varicella to the genuine and true Small-pox, as merely so many modifications, or transitionary conditions, of the same disease. The variolous affections of the lower animals were quite unknown to the Greek and Roman writers; and, even for some centuries after the disease in man had been widely diffused through Europe, they seem to have escaped notice. In Asia, however, where the human disease has been for ages known, they have been long recognised and understood. Even in Mexico, according to the testimony of Humboldt, the natives were quite aware that the variolous affections of brutes were transmissible to mankind, and had the effect, when so communicated, of protecting the individuals against the small-pox.

M. Heusinger is of opinion that the following conclusions are fairly warranted by historical evidence. 1. That the small-pox did not originate in brutes, but that it has been communicated to them from man. 2. That the variola is transmissible from one species of animal to another, from man to the brutes, and from them to him. 3. The more fully that the disease is developed, the more completely is the affected individual, whether human or brute, protected from a second infection; and *vice versâ.*—The best writers on the effects of inoculating different animals with the variolous poison are Sacco, Numan, and Ceeley. It is strange that so recent an author as M. Dupuy should have adopted the old error of seeing nothing but a Variolous disease in all Epizootics.*

The different modifications of variolous eruption, as observed in different species of animals, may be enumerated thus;—1. *Variolæ ovillæ, schafpocken, la picote, le claveau, la clavelée.* When fully developed, it is very like the genuine small-pox eruption in man; but in sheep, as in the human species, there are various degrees or forms of the disease. Godine and others have infected sheep by inoculating them with human virus; and Sacco asserts that he has transplanted the disease called *la clavelée* upon man, and succeeded in thus producing a genuine protective vaccination.—2. *Variola Caprina.* Valentin and Numan have satisfactorily shown that the vaccine virus is readily communicable to goats: we may, therefore, presume that these animals often suffer from the variolous poison.—3. *Variola Canina.* It is a tolerably frequent epizootic among dogs, and resembles a good deal the disease as it is seen in sheep. Cases are recorded where the dog has been infected from the human being, as well as from sheep labouring under the *clavelée.*—4. *Variola Suilla.* The disease has been long known among swine; and it has often been observed to prevail among these animals at the same time that there was an epidemic of small-pox, and an epizootic of the *claveau* among sheep. Viborg alludes to the successful inoculation of swine with human variolous matter.—5. *Variola Vaccina* was known anciently in Asia, and for a great length of time in Southern America, as well as in various districts of Europe: to medical men generally, only since the commencement of the present century. Some writers are of opinion that an epizootic of Vaccinia may become developed in the cow primarily; i. e. independently of external contagion. But it may be fairly remarked, in reference to this point, that the sources of contagion are often exceedingly obscure; the history of the malady, and the various experiments upon the subject of inoculation in different animals, would rather lead us to believe that the vaccine disease is in truth the human small-pox transmitted to the

* Traité sur les Maladies Epizootiques. Paris, 1837.

cow, either directly or indirectly, through the medium of sheep, dogs, horses, &c.

From the remotest period, it has been well known in Asia that the cow-pox often infected human beings, and that individuals so infected were exempt from small-pox; indeed, this fact had been repeatedly observed in the different countries of Europe for a full century before Jenner directed public attention to the important circumstance. The vaccine virus has been transmitted to all other domestic animals, and has been found to protect them, as well as man, from their variolæ.—6. *Variola Equina.* The virus of vaccinia, and also of human variola, has been inoculated upon the horse; and the disease, so produced, when transplanted upon other animals or upon man, has exhibited all the characteristics of genuine vaccinia. Jenner maintained that the cow-pox was communicated to cows by horses affected with the disease called "grease." This opinion was, ere long, shown to be far too exclusive; many experimental inoculations failed entirely; and subsequent observations have proved both the spontaneous transmission, and the possibility of the inoculation of the grease upon man, and the lower animals; and that the disease, so induced, was altogether like cow-pox. We may, therefore, fairly assert that there is really and truly a *variola equina* occurring in the feet of horses; although unquestionably more diseases than one have been comprehended under the appellation of the "grease," "eaux aux jambes," and "mauke," (German).

Before dismissing the subject of Variola, we may mention that it has been long known in the East that the Camel is liable to be affected with an Eruption like Cow-pox, and that it is communicable to mankind.*

*Pustula Maligna, Anthrax Epizooticus, Milzbrand, &c.* is perhaps the most general disease of all that occur among animals. Not only our domestic tribes, but even the wild animals of the forest, are liable to be affected with it. Sometimes it occurs sporadically; at other times it prevails as an enzootic or epizootic malady: it is eminently contagious. * * * * If the disease, remarks our author, arises spontaneously in the horse, cow, sheep, or sow, more especially if it assume an acute form, there is often no formation of pustules or carbuncles; in other cases, indeed, these local phenomena manifest themselves very soon after the commencement of the constitutional malady. When, however, the disease arises from the immediate contact of the blood of an infected animal, it always commences in the local formation of a pustule or anthrax.

M. Heusinger tell us that the disease is enzootic in a valley of his country, and that the inhabitants of the district are unusually liable to carbuncular affections. The genuine *pustula maligna* very rarely arises spontaneously in the human being; it is almost always communicated from an infected animal. He has only seen two cases where the disease was of primary development in man; and in both cases, the persons affected

---

* Masson's Narrative of a Journey to Kelat.—Transactions of the Medical and Physical Society of Bombay, 1840. P. 214.

had been engaged in the soaking of flax (travail dans les routoirs de lin). It is a curious circumstance that the few analogous cases, that have been recorded by other writers, have been attributed to a similar cause. The malignancy of the disease, when it occurs in man, appears to be in some measure dependent upon the species of animal from which the infection has been derived. When received from the horse it is always, from the cow it is often, very dangerous; whereas, when received from the sheep, it is generally slight, and sometimes only local. M. Heusinger has never known a case where the disease was communicated from one human being to another. There cannot be a reasonable doubt that the essential nature of the disease is still unknown; all that we can safely assert is, that there is induced a decomposition or morbid alteration of a peculiar character of the circulating fluids.

We cannot find space to notice, however briefly, various other cutaneous affections that are sufficiently common among the lower animals. We must also pass over the diseases of the hoofs; especially as it would be difficult to point out any close analogy with the morbid conditions of the corresponding parts in the human subject, in consequence of the marked difference in the organization. We may remark that many ungular diseases are apt to become contagious, more especially in horses and sheep.

*Hygromata*, or dropsical swellings of the *bursæ mucosæ*, are more common in the horse than in man: they are most frequently observed on the backs of those animals, which are made beasts of burden. In the camel, they sometimes acquire a very large size. The *Elephantiasis Arabum*—pes elephanti—is often met with in the horse.

We now pass on to notice some of the Diseases of the Urinary Organs.

*Diabetes*, or *Hydruria*.—This is a common complaint in horses. Hitherto no saccharine matter has ever been detected in the urine of these or of any of the inferior animals. In an epizootic diabetes which prevailed some time ago at Paris, M. Lassaigne discovered in the urine of many horses a notable quantity of free acetic acid*—a circumstance that is rather remarkable. In reference to this Epizootic, it also deserves to be noticed that it was almost exclusively among *entire* horses that the disease was observed to prevail; very few geldings, and not one mare, were affected, according to the observations of M. Moiroud. Now, in the human subject, *melituria* is very rarely met with among females, or before the age of puberty. Hydruria or diabetes insipidus, however, is more common in women than among men; and M. Greve asserts that the ordinary hydruria in horses is most frequent among geldings, and least so among stallions. The whole subject, however, requires to be examined with care, before we can attach much importance to any statements touching the relative frequency of the disease. *Albuminuria* has been observed in the horse by Messrs. Clayworth and Percivall. *Hæmaturia* is rare among

* The urine of horses, and indeed of herbivorous animals generally, in a state of health, has an alkaline re-action. Hence it quickly becomes ammoniacal, and emits a peculiar penetrating odour. After it has rested for a time, it is often so tenacious and viscid that it may be drawn up in long threads.

dogs; more frequent in horses and swine than in man; and still more so in cows and sheep; among these last-named animals, the disease is sometimes observed to be enzootic and epizootic. We must distinguish the hæmaturia arising from the irritation and inflammation of the kidneys, that is apt to be caused by the swallowing of certain insects and acrid vegetables, from the cachectic species of the malady that is connected with a dissolved state of the blood, or great debility of the renal structure. *Lithiasis*, or the formation of gravel or calculi in the kidneys and bladder, is of very frequent occurrence in our domestic animals. Generally speaking, the constituents are much the same as in man; except that no uric acid occurs in the calculi of the herbivora, which consist, for the most part, of earthy phosphates and carbonates. The quantity of carbonate of lime is sometimes very large. Hitherto but little attention has been paid to this subject by veterinary surgeons, so that we are unable to say whether the disease be Enzootic in those districts where it may be said to be Endemic. In many of the rodentia, the presence of sedimentary matters in the urine seems to be almost natural; it is of such common occurrence.

There are none of the diseases of the sexual organs in the male animal that call for notice.

### Diseases of the Uterus.

*Puerperal Metritis* (kalbefieber, milchfieber) is perhaps as common among the lower animals as among women: Oopharo-metritis is also the most frequent form of the disease: the affection of all the serous membranes, and especially of the pia mater, is a common complication. If, on the one hand, veterinary practitioners have not sufficiently distinguished all the forms of the malady—for example, uterine phlebitis—it must be admitted, on the other hand, that they have not allowed themselves to be so much led away by merely fanciful speculations, as some of the recent writers on puerperal fever in Germany have done.

*Uterine Polypi* are frequent in the mare and cow.—*Scirrhous and Cancerous degeneration* of the womb* has been seen in the cow and bitch.—*Prolapsus uteri* is often met with in our domestic animals; and rupture of this organ is not very unfrequent.

The *Mamma* or teat in animals is in them, as in women, not unfrequently the seat of scirrhous and carcinomatous disease. The alterations of the milk, in various morbid conditions, appear to be very much the same in both.†

* Dr. Walshe, in his very elaborate work on Cancer just published, quotes from our author various data respecting the occurrence of this disease in the lower animals. In his enumeration of the different parts of the body in which it has been observed in the cow and bitch, Dr. W. has omitted (it must have been from mere inadvertence) to mention the *uterus*.

† M. Dumas has recently published some analyses of the milk of bitches, with the view of shewing that, after they have been kept for some time upon a

M. Heusinger remarks, that the treatment of milk-swellings (milchknoten) is generally much better understood by veterinary surgeons than by practitioners in human medicine.

For want of space, we must pass over any mention of the diseases of the muscular and osseous systems; although there are many points, especially in reference to the latter, that might justly have claimed our attention. The frequency of *exostosis* in some animals, especially among poultry, and the not uncommon occurrence of *osteo-malacia* in others, as among the monkeys of our menageries, are curious circumstances.

### Cachexiæ.

Unquestionably the cachectic diseases of the lower animals exhibit many points of marked difference from those that are observed in the human subject; but it is equally certain that not a few traits of resemblance—and this too of a practically important nature—may be easily discovered between them. The grand object alike of the human and veterinary practitioner ought to be to seek to find out their inducing causes, with the view of preventing and curing the vitiated condition of the fluids that is present. This may be a proper opportunity to mention that M. Andral, as well as some other French pathologists, have recently been examining the condition of the blood of animals when labouring under different diseases.

"From 27 analyses of the blood of 11 sheep affected with the watery cachexia or the rot, it would appear that the amount of fibrin is slightly affected, but that the blood-corpuscles are excessively diminished; from 78, their normal average, they fall to 40, 25, and even 14. The solid residue of the serum is diminished, (a point in which this disease differs from chlorosis in the human subject), and the water is considerably increased.

"The deficiency in the amount of blood-corpuscles appeared to vary with the progressing weakness of the animal. By proper food, and due attention to atmospheric influences, the corpuscles were observed to increase; in one instance they rose from 49 to 64.

"From 14 analyses of the blood, in which this affection was associated with inflammatory disorders, it appeared that the fibrin increases, and the blood-corpuscles diminish, as in simple, uncomplicated inflammations."

These observations, we may remark, are derived from Dr. Day's translation of Simon's Animal Chemistry. We now proceed to notice very briefly some of the Cachectic diseases enumerated by our author.

The *cachexia agnosa vel hydropica*—characterised by impoverishment of the blood, atrophy of all the tissues, and accumulation of serum in the cellular tissue and sacs of the body—is common to all our domestic animals with man. It is often observed to be endemic and enzootic, epidemic and epizootic, occurring in the same districts, at the same periods, and under the same conditions.

---

purely animal diet, all traces of sugar disappear from their milk.—*Annales des Sciences Nat.* Sept. 1845.

*Scrofula*, too, is of very frequent occurrence among all animals, birds as well as mammals. It occurs under the same physical circumstances and influences, which are known to promote its development among human beings. It is most frequently enzootic; very often it is hereditary. Herbivorous animals are much more frequently affected with it than carnivorous.

The following instance will serve to illustrate very well the transition of different forms of scrofulous diseases, from one to the other.

A flock of 350 lambs was kept in a warm, ill-ventilated, stable, and fed with over-rich and succulent food. In the course of a very short time, they were covered with blotches of a pustular eruption. When this was found out, the animals were injudiciously turned out into the fields, exposed to the cold air, and where the pasture was very poor. The almost immediate effects were that the cutaneous disease indeed disappeared; but, in its place, a sort of catarrh, followed by a mesenteric atrophy, set in, and carried off a multitude of the animals.

Different forms of scrofulous disease are observed to be predominant in different tribes of the lower animals. Thus, in the horse, it is the pituitous membrane that is generally affected; in sheep, the spine, producing rhachitis; in the cow, the skin; and in the sow, the glandular tissue.

Authors are not yet agreed upon the somewhat important point, whether the common diseases of Glanders and Farcy are to be regarded as forms or varieties of Scrofulous cachexy or not. Without presuming to decide upon this question, we shall merely introduce a few descriptive remarks at present.

The *farcy* consists in a hypertrophy, dilatation, inflammation, and ulceration of the lymphatic vessels and glands of the skin; the tumours and ulcers, that are formed, resembling very closely the scrofulous tumours and ulcers which occur upon the skin in human beings. The disease is sometimes developed in adult animals under the same set of influences which are apt to induce scrofula in young ones; very frequently, however, it is the result of contagion. It is common in the horse; rare in the cow; and has never been observed in any other of our domestic animals. M. Renault thinks that he has shewn that the development of Farcy arises from absorption of pus by the lymphatic vessels; but his observations admit of another explanation. MM. Hamont and Prunner, who have had extensive opportunities in the East of observing the two endemic and enzootic diseases of Elephantiasis Græcorum or Lepra tuberculosa in man, and of Farcy in the horse, maintain that they are identical in their nature. That there are many points of resemblance between them, cannot be doubted; but the writings* of these authors unquestionably contain many most erroneous statements. The disease in man, which appears to come the nearest to the equine malady, is that which Autenreith has called "gonorrhœal scrofula" (tripper-scrofeln).

*Glanders* (la morve, der rotz) is justly regarded by most observers as

---

* Journal de Medecine Veterinaire Theor. et Prat.: Vol. V. p. 193.

merely a form or variety of the Farcy. Its essential symptoms are—engorgement and induration of the lymphatic glands of the throat, a purulent discharge and specific ulcers (not unlike those of a syphilitic origin) of the nasal membrane; at length, erosion and destruction of all this membrane, and caries of the nasal bones: the animal dies phthisical, if he is not more quickly cut off by phthisis, general inflammation of the lymphatic vessels, or gangrene of the lungs. The disease is frequently complicated with genuine Farcy. It arises, primarily and spontaneously in equine animals alone; more frequently in the ass and mule than in the horse itself: for the malignant Catarrh of the cow and sheep, and the distemper of dogs, present many points of marked difference. The causes are the same as those of Farcy; the virus too of the one disease sometimes produces the other malady, and *vice versâ*.

In connexion with some of the preceding remarks on Glanders and Farcy, it is worthy of especial notice that, in all scrofulous constitutions, there is a marked tendency to one or more of the mucous membranes being the seat of suppuration and ulceration; the surface of the air-passages more particularly so. Doubtless, the greater number of scrofulous affections are not of a contagious nature. Some, however, occasionally seem to be so; for example, Blenorrhœa of the eyes and lungs. Perhaps, indeed, we may go a step further, and say that there is scarcely any malady that may not, under certain circumstances, acquire a contagious property.

"It is singular," M. Heusinger remarks, "that I have never seen an instance of Glanders in the human subject, although every year cases of poisoned inflammation of the skin, caused by the absorption of an animal virus and by that of the glanders itself, are presenting themselves to my notice. During the year 1817-18, fully one-third of the horses of our ambulance died of this disease. I dissected many of the animals, and I made numerous experiments upon them, without taking any particular precautions to protect myself against infection. Subsequently to this period, my observation of the disease in horses has been very extensive; yet, as I have said before, I have never witnessed a case of it in the human subject."*

Our author enumerates (with a good deal of propriety, in our opinion) among the Cachexiæ, a "c. arthritica" and a "c. rheumatica;" regarding the diseases of Gout and Rheumatism, as depending upon a morbid condition of the fluids.

*Rheumatism.*—Rheumatic affections are exceedingly common in all our domestic animals. The disease, which has been called *coriago, echedermia, harthautigkeit,* is a rheumatic affection of the cellular sheaths of the skin and panniculus carnosus.—Articular rheumatism of the extremities, and of the vertebral column, is often the cause of exostosis and anchylosis.—Rheumatic pericarditis is probably much more common in the lower animals than is generally imagined.—Rheumatic *meningo-myelitis* is a disease that is more frequent and more fatal among animals than among human

---

* There is a series of valuable papers on Glanders and Farcy by Mr. Percivall, as well as occasional contributions by other writers, in the Veterinarian for 1845. M. Heusinger makes frequent reference to this valuable periodical.

beings: cows and oxen are those most frequently affected with it. This disease should be carefully distinguished from mere rheumatism of the spinal column. Dogs are very subject to rheumatism, especially of the spine, and to a permanent paralysis of the hinder extremities induced thereby.—Veterinary practitioners are not completely agreed among themselves whether genuine arthritis or gouty inflammation is ever met with in animals.

The existence of the proper *Scorbutic cachexy* among the lower animals has not hitherto been very clearly made out. There is good reason to suspect that it does occur; but the observations, which have been recorded, are not very satisfactory; and some of the maladies, which have been described under the appellation of scorbutic, were certainly of another character. Several of the French and German writers have confounded carbuncular with scorbutic affections. According to our author's experience, animals are liable to be affected with the "pourriture" in those seasons, when scurvy prevails among the inhabitants of the district.

*Cachexia Syphilitica.*—On this subject M. Heusinger declares that, from the most remote period, human beings were subject to contagious discharges, vegetations and ulcers of the sexual organs; that these were communicable by coitus, and often occasioned a general cachexia; that there were similar vegetations of the skin in warm climates, which indeed have not usually been regarded as of a syphilitic origin, but which are probably so nevertheless; and lastly that, about the close of the 15th century, under the concurrence of epidemic influences, the form of the disease now known became developed. It is well known to all observant practitioners that, even in the present day, epidemic agencies exert a marked influence upon the development of syphilitic maladies: in some years, they are exceedingly common and very intractable; while, in others, they are mild and much less frequent. Again, in one season almost every patient is affected with gonorrhœa; in another, vegetations or ulcerations are the predominant affection.

All our domestic animals are subject to contagious discharges and ulcerations of the generative organs: they are transmissible by coitus, and exhibit a resemblance to syphilitic affections; although they certainly do not proceed from them, as some writers have imagined. They have been described under the names of Syphilis, Frambœsia, &c. In my opinion, says M. Heusinger, they are nearly in the same condition as the syphilitic affections of man were before the close of the 15th century.

### Diseases of the Nervous System.

*Hydrencephalitis* is a disease that is known in animals by the name of "acute vertigo." It is more frequent in the horse than in sheep or oxen. The chronic form of Hydrocephalus is of more frequent occurrence than the acute, especially in the horse. Cerebral *congestion* occurs very frequently in all our domestic animals. Cerebral hæmorrhage, although less common than in man, is not a very rare affection among them. Morbid affections of the Spinal Marrow are common in most of our tame animals. M. Bouley has written an admirable paper upon this branch of comparative pathology—(*Recueil de Med. Veterinaire,* t. vi.)

*Hydrorachis*, or dropsy of the spinal-marrow, occurs much more frequently among many of the lower animals than in our own species. It is often congenital among lambs. An effusion of purulent matter within the spinal canal has been repeatedly found in horses. Inflammatory *ramollissement* too of the medulla is not an unfrequent affection.

*Paralysis* is a common disease among brutes. A singular epizootic malady of a paralytic nature is that which in Germany was called *sterzwurm* or *sterzseuche*, but which now appears to be scarcely known to veterinarians in the present day. It has been very improperly designated *epizootia Cancrosa* by Ampach, and regarded by him as being of a gangrenous nature. The gangrene of the tail, that takes place, is only a secondary, and not a primary or essential, symptom. From the descriptions that have been given of the disease, we may suppose that it was a (perhaps rheumatic) paralysis of the tail.—*Tetanus* is a disease that is singularly frequent in horses : it is of rare occurrence among other domestic animals. *Epilepsy* is most frequently observed among dogs, cats, and birds: *Chorea* also has been witnessed in dogs.

*Rabies*, as far as we know, always originates in the canine species, the dog, the fox, the wolf, or the jackall. Some writers, however, have been of opinion that it may originate in the cat. The disease is transmissible to all other quadrupeds, and even to birds. It is doubtful whether it is ever propagated by animals that have become infected, but in which it is not of original development. We possess no satisfactory evidence that it has ever been communicated from one human being to another.

In taking leave of M. Heusinger for the present (for it is possible that we may recur to his work, when it is completed), we cannot but again express our admiration of his marvellous industry, great erudition, and extensive scientific acquirements. These Researches should be in every public library throughout the kingdom : they are a mine of curious and instructive information.

